# Characterization of lncRNA–miRNA–mRNA Network to Reveal Potential Functional ceRNAs in Bovine Skeletal Muscle

**DOI:** 10.3389/fgene.2019.00091

**Published:** 2019-02-20

**Authors:** Binglin Yue, Hui Li, Mei Liu, Jiyao Wu, Mingxun Li, Chuzhao Lei, Bizhi Huang, Hong Chen

**Affiliations:** ^1^College of Animal Science and Technology, Northwest A&F University, Yangling, China; ^2^State Key Laboratory for Conservation and Utilization of Subtropical Agro-Bioresources, Guangxi University, Nanning, China; ^3^College of Animal Science and Technology, Yangzhou University, Yangzhou, China; ^4^Yunnan Academy of Grassland and Animal Science, Kunming, China

**Keywords:** bovine, ceRNA, lncRNA, skeletal muscle, enrichment analysis

## Abstract

There is growing evidence that non-coding RNAs are emerging as critical regulators of skeletal muscle development. In order to reveal their functional roles and regulatory mechanisms, we constructed a lncRNA–miRNA–mRNA network according to the ceRNA (competitive endogenous RNA) theory, using our high-throughput sequencing data. Subsequently, the network analysis, GO (Gene Ontology) analysis, and KEGG (Kyoto Encyclopedia of Genes and Genomes) pathway analysis were performed for functional annotation and exploration of lncRNA ceRNAs. The results uncovered a scale-free characteristics network which exhibited high functional specificity for bovine skeletal muscle development: co-expression lncRNAs were significantly enriched in muscle development related biological processes and the Wnt signaling pathway. Furthermore, GSEA (Gene Set Enrichment Analysis) indicated that the risk score has a tendency to associate with myogenesis, and differentially expressed RNAs were validated by qPCR, further confirming the credibility of our network. In summary, this study provides insights into lncRNA-mediated ceRNA function and mechanisms in bovine skeletal muscle development and will expand our understanding of lncRNA biology in mammals.

## Introduction

Vertebrates’ skeletal muscles are mostly derived from paraxial mesodermal somites and undergo hyperplasia and hypertrophy process successively (Buckingham et al., 2003; Guo et al., 2015). Vertebrates’ skeletal muscle development is a complex process, which plays a crucial role in overall body metabolism, and a transcriptional hierarchy including the MRFs (myogenic regulatory factors) and MEF2 (myocyte enhancer factor 2) family precisely orchestrates it through coordinating the activities of a series of muscle genes (Pownall et al., 2002; Buckingham, 2006). Furthermore, several studies showed that post-transcriptional regulation also has a significant effect on skeletal muscle development (Cesana et al., 2011). However, the precise molecular mechanisms are still poorly understood (Te et al., 2010; He and Liu, 2013).

NcRNAs (non-coding RNAs) are RNA molecules that have little protein-coding capacity, but rather than being transcriptional noise, serves as master regulators in a variety of biological processes (Huarte, 2009; McHugh et al., 2015). Recently, accumulating evidence has suggested that ncRNAs like miRNA (microRNA) and lncRNA (long non-coding RNA) are emerging as critical regulators of skeletal muscle development (Williams et al., 2009; Matsumoto et al., 2017). Like mRNA-like transcripts longer than 200 nucleotides, lncRNAs have extremely abundant binding sites for microRNAs (Phelps et al., 2016), and LncRNAs such as linc-MD1 (Cesana et al., 2011), H19 (Kallen et al., 2013), lncMyoD (Gong et al., 2015) and lnc-mg (Zhu et al., 2017) showed relevant roles in myogenesis by acting as ceRNA (competing endogenous RNA). The ceRNA function, how noncoding RNAs and mRNAs regulate each other by competing for binding to shared miRNAs using partially complementary sequences known as MREs (miRNA response elements), which induces degradation of mRNA targets or translation repression at the post-transcriptional level (Salmena et al., 2011). Up to now, only few lncRNAs have been functionally well annotated (Butchart et al., 2016; Yu et al., 2017). Considering this, the aim of this study was to further identify a novel regulatory mechanism based on the ceRNA theory in bovine skeletal muscle development.

**FIGURE 1:**
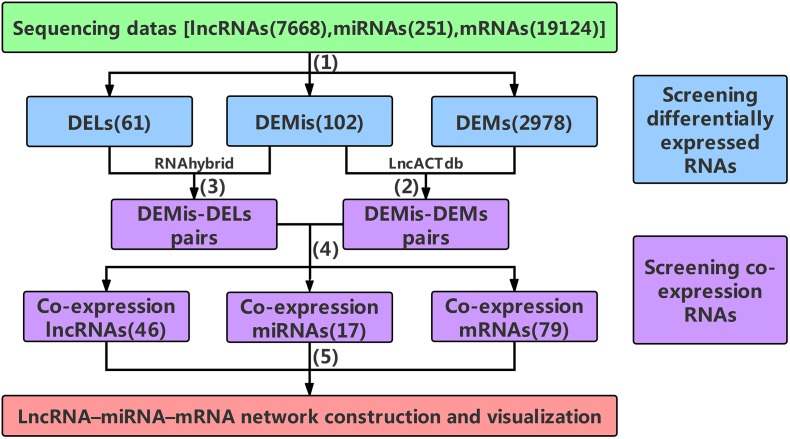
The main frame of data processing. **(1)** High-throughput sequencing data were pre-selected according to the | log_2_FC| ≥ 4(| log_2_FC| ≥ 2 for DEMs) and *P*-values < 0.001. **(2)** The DEMis-DEMs pairs were obtained using LncACTdb. **(3)** According to MFE theory, the DEMis-DELs pairs were screened using the RNAhybrid program. **(4)** the DEMis-DEMs pairs were merged with the DEMis-DELs pairs to obtain the co-expression RNAs. **(5)** 46 co-expression lncRNAs, 17 co-expression miRNAs, and 79 co-expression mRNAs were finally selected to construct the lncRNA–miRNA–mRNA network. DELs, differentially expressed lncRNAs; DEMis, differentially expressed miRNAs; DEMs, differentially expressed mRNAs; MFE, minimum free energy.

In recent years, RNA-Seq (RNA sequencing) based on high-throughput platforms has emerged as an efficient tool to screen genomic variation, associated with divergent phenotypic traits and identifies the molecular mechanisms underlying the domestication process (Dong et al., 2015). In the present study, our high-throughput sequencing data were processed to construct the lncRNA–miRNA–mRNA network, which has the potential to highlight specific molecular functions and mechanisms (Sun et al., 2013, 2016; Li et al., 2017; Figure 1). Afterward, functional enrichment analyses and annotation were performed to further explore the importance of lncRNAs in myogenesis. Considering this, our study expands the potential lncRNA ceRNA functions during bovine skeletal muscle development and enhances the understanding of non-coding RNA regulatory networks.

## Results

### Processed-Data Analysis

**FIGURE 2:**
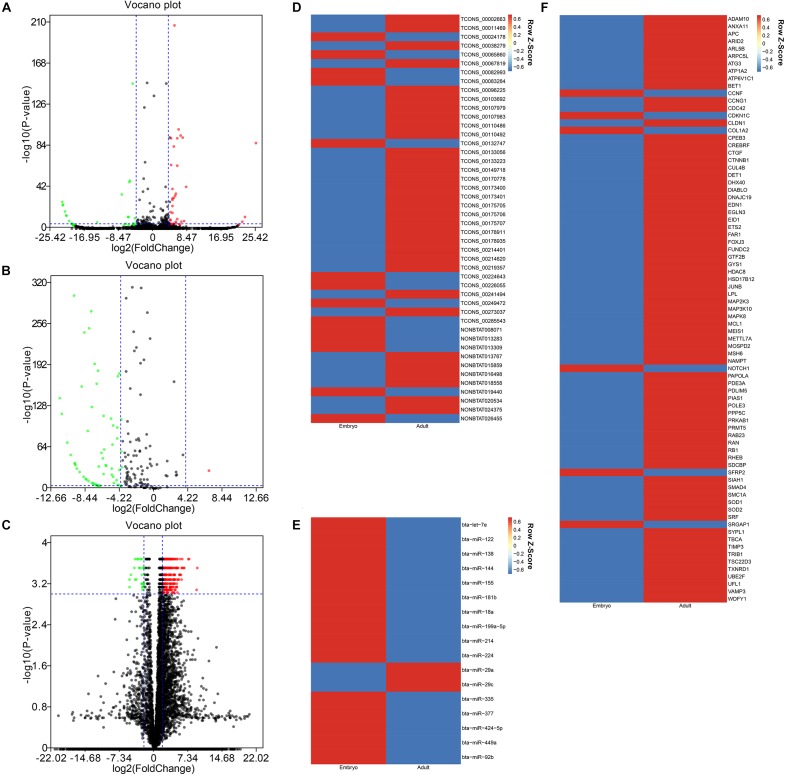
The differentially expressional pattern of RNAs from bovine embryonic and adult stages. **(A-C)** Volcano plot of 61 DELs **(A)**, 102 DEMis **(B)**, and 2978 DEMs **(C)** for embryo versus adult. Each point indicates an RNA. Green dots denote down-regulated RNAs, red points represent up-regulated RNAs under the same thresholds, and black points indicate RNAs that did not change significantly. The criteria is based on| log2FC| ≥ 4(| log2FC| ≥ 2 for DEMs) and *P*-values < 0.001. **(D-F)** Heatmap of 46 co-expression lncRNAs **(D)**, 17 co-expression miRNAs **(E)**, and 79 co-expression mRNAs **(F)** displaying when comparing embryonic and adult stages. Each column represents one sample, and each row refers to an RNA. The color legend is on the top-right of the figure, and compound relative abundances were standardized (*Z* score, shown in legend) prior to unsupervised hierarchical clustering of samples (rows). Blue indicates RNAs with a lower expression relative to the geometrical means; red indicates genes with a greater expression relative to the geometrical means.

After data was processed, a total of 61 DELs (differentially expressed lncRNAs), 102 DEMis (differentially expressed miRNAs), and 2978 DEMs (differentially expressed mRNAs) were identified between two developmental stages in Chinese Qinchuan bovine longissimus muscles. Among these differentially expressed RNAs, DEMis were mostly down-regulated in samples from the embryonic stage to the adult stage, while DELs and DEMs showed the opposite results (Figure 2A–C). As described in the Materials and Methods section, we identified 46 co-expression lncRNAs, 17 co-expression miRNAs, and 79 co-expression mRNAs, which were selected to construct the lncRNA–miRNA–mRNA network (Figure 2D–F).

### LncRNA–miRNA–mRNA Network Construction and Visualization

**FIGURE 3:**
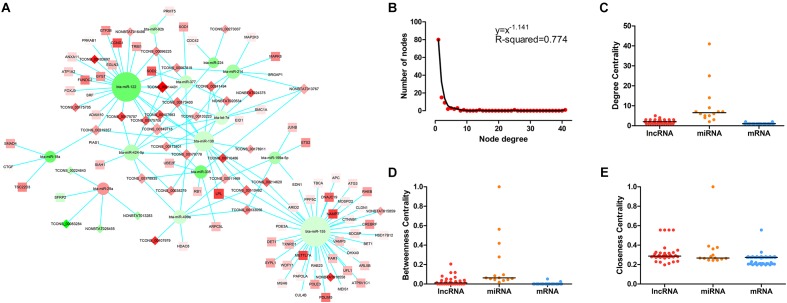
The LncRNA–miRNA–mRNA network and its structural characteristics. **(A)** Global view of the network in bovine skeletal muscle development. This network consists of 141 edges among 35 lncRNA nods (rhombus), 14 miRNAs (circle), and 69 mRNAs (square). Red nodes represent upregulation, whereas green nodes represent downregulation. The node size typifies the level of edges enrichment: the higher the degree, the bigger the size is. **(B)** Degree distribution of the network. **(C)** The difference of degree centrality among lncRNAs, miRNAs, and mRNAs. The lncRNA nodes had a significantly higher degree centrality than mRNA nodes in the network. **(D)** The difference of betweenness centrality among lncRNAs, miRNAs, and mRNAs. The lncRNA nodes had a higher betweenness centrality than mRNA nodes in the network. **(E)** The difference of closeness centrality among lncRNAs, miRNAs, and mRNAs. The lncRNA nodes had a higher closeness centrality than mRNA nodes in the network. *P*-values were calculated based on Kruskal–Wallis test.

The LncRNA–miRNA–mRNA network was constructed on the basis of these co-expression RNAs and visualized using Cytoscape. As shown in Figure 3A, 35 lncRNA nodes, 14 miRNA nodes, 69 mRNA nodes, and 141 edges composed the lncRNA–miRNA–mRNA network. In the study of networks, the degree of a node indicates the number of edges linked to the given node, and the degree distribution is the probability distribution of these degrees over the whole network. Following the network analysis, indicators of centrality such as DC (degree centrality), BC (betweenness centrality), and CC (closeness centrality) exist to determine the importance of a single node which possesses essential functions in a complex network. The degree of distribution of nodes in the network was investigated, and the power-law distribution with a slope of –1.141 and R-squared = 0.774 was observed (Figure 3B), suggesting that the network displayed scale-free characteristics typical of biological networks. Further comparison analysis then showed that there were significant differences in the DC, BC, and CC among lncRNAs, miRNAs, and mRNAs (*P*-value < 0.01 for DC, BC, and CC using Kruskal–Wallis test) (Figure 3C–E), indicating that lncRNAs and miRNAs tended to be critical in the context. Overall, the scale-free distribution and module characteristics of the entire network implied the presence of functionally important nodes in bovine skeletal muscle development.

### Molecular Function and Pathway Prediction

**FIGURE 4:**
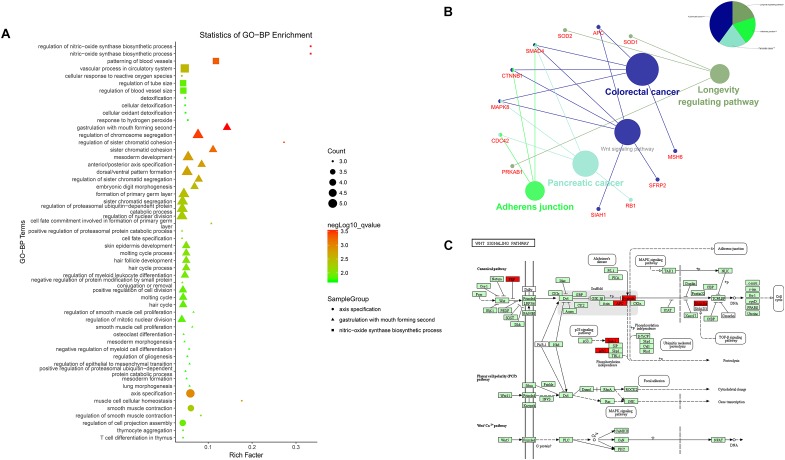
Enrichment analysis of differentially expressed mRNAs. **(A)** Advanced bubble chart of GO-BP. Y-axis label represents terms name, and X-axis label typifies rich factor which is defined as the percentage of target genes per term. Size and color of the bubble are measured as count and –log_10_qvalue to represent the amount of differentially expressed mRNAs enriched in these terms and the enrichment significance, respectively, as well as three shapes of SampleGroup represent three functional clusters. **(B)** KEGG interaction network. Terms are represented as nodes based on the kappa score level (= 0.4), and node size indicates the term enrichment significant. Edges represent shared genes, the wider the edge, the larger the overlap is. The colors indicating different functional groups are shown at the right top. **(C)** Bos taurus Wnt signaling pathway map (Kanehisa et al., 2017). Upregulated genes in our network are marked in red.

Results of the GO analysis revealed 54 BP (Biological Process) terms (Supplementary Table S1) which were mainly involved in three functional clusters, including nitric-oxide synthase biosynthetic process, gastrulation with mouth forming second, and axis specification. BP terms like muscle cell cellular homeostasis, mesoderm development, axis specification, and dorsal/ventral pattern formation were found in the enrichments, indicating the regulatory roles of these co-expression lncRNAs in bovine skeletal muscle development (Figure 4A). Following this, a pathway analysis demonstrated that these co-expression mRNAs were enriched in five pathways, such as longevity regulating pathway, adherens junction pathway, pancreatic cancer pathway, colorectal cancer pathway, and Wnt signaling pathway (Supplementary Table S2, Figure 4B), in which the Wnt signaling pathway is essential for embryonic muscle development and maintenance of adult skeletal muscle homeostasis (Figure 4C; Kanehisa et al., 2017). For instance, SFRP2 appears to function as a Wnt antagonist to prevent myoblasts from entering the terminal differentiation process in embryonic myogenesis (Ladher et al., 2000). As a downstream effector of the canonical Wnt signaling pathway, β-catenin (*CTNNB1*) affects muscle mass and slows fiber numbers in mice with conditional deletion of *CTNNB1* in the muscle progenitor cells (Hutcheson et al., 2009). In addition, Wnt signaling activation was also suggested to induce satellite cell proliferation during skeletal muscle regeneration (Otto et al., 2008). These results suggested that the LncRNA–miRNA–mRNA network is related to skeletal muscle development.

### GSEA Increased the Credibility of the LncRNA–miRNA–mRNA Network

**FIGURE 5:**
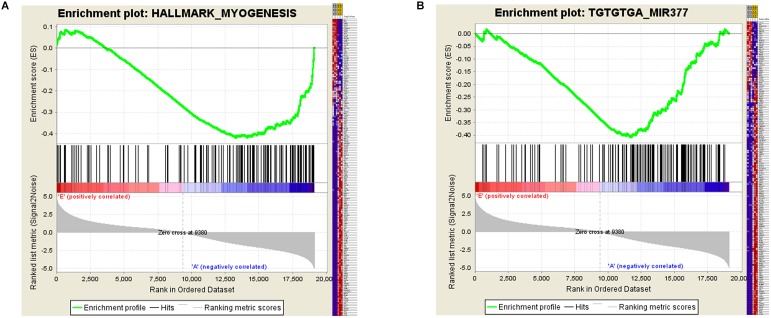
Gene Set Enrichment Analysis (GSEA) of mRNA expression levels in bovine skeletal muscle. **(A** and **B)** GSEA result using HALLMARK_MYOGENESIS **(A)** and C3 MIR gene sets **(B)** Left panel: X-axis label represents “E”(Embryonic stage)/“A”(Adult stage) level, and Y-axis label represents the enrichment score of these mRNAs. Right panel: heat map of related mRNAs expression in two stages.

Certain gene sets of the HALLMARK_MYOGENESIS from MSigDB, previously described to be involved in myogenesis, were used in Gene Set Enrichment Analysis (GSEA), and the adult high expression showed a tendency for a high enrichment score of the myogenesis-related gene set (NES: –1.83, FDR: 0.0003) (Supplementary Table S3 and Figure 5A). Moreover, the miR-377 targets were shown to be significantly located in the bottom of the pre-ranked gene-list in adult high expression (NES: –1.76, FDR: 0.006) by subjecting C3 MIR gene sets (Figure 5B), in which the targeted relationship of miR-377-SOD1 was predicted in the LncRNA–miRNA–mRNA network (Supplementary Table S4). These results increased the credibility of the LncRNA–miRNA–mRNA network to a certain degree.

### Validation of Differentially Expressed RNAs by qPCR

**FIGURE 6:**
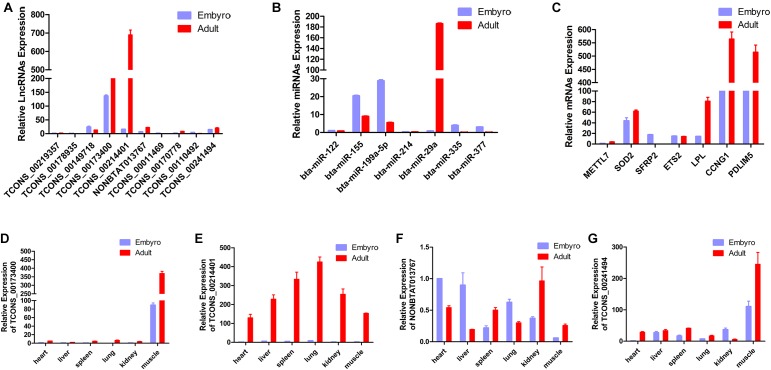
Validation of putative RNAs by quantitative real-time PCR. **(A)** Absolute quantification for 10 lncRNAs in longissimus muscle tissue from the fetal and the adult Qinchuan cattle. **(B)** The expression of 7 miRNAs in longissimus muscle tissue of Qinchuan cattle at the embryonic stage compared with the adult stage. **(C)** The expression levels of 7 mRNAs in longissimus muscle tissue from the fetal and the adult Qinchuan cattle. (**D-G)** The expression of 4 randomly selected lncRNAs in different tissues from the fetal and the adult Qinchuan cattle. Values are means ± SEM for three individuals. *P*-values < 0.05.

In the present study, a total of 10 lncRNAs, 7 miRNAs, and 7 mRNAs were randomly selected from the constructed network to perform validation by qRT-PCR. We confirmed stage-specific differences in the abundance of certain RNAs when comparing embryonic and adult longissimus muscle tissue samples using qPCR. qPCR expression patterns of the 10 lncRNAs, 7 miRNAs, and 7 mRNAs were basically similar to high-throughput sequencing results, which verified the reliability of sequencing results (Figure 6A–C). In order to explore the function of lncRNAs in myoblasts, 4 lncRNAs were selected to perform the qPCR for different tissues from different stages. We found that these lncRNAs had high expression levels in muscle tissues, and their expression was much higher at the adult stage compared to the embryonic stage (Figure 6D–G). These results implied their regulatory roles in muscle development.

## Discussion

The development of bovine skeletal muscle is a complex process involving prenatal and postnatal patterns, which are mainly due to all muscle fiber formation, the muscle fiber size increases and new muscle fibers regenerate, respectively (Buckingham et al., 2003). In our research, two developmental stages were chosen: the embryonic 90-day stage, a prenatal generation when a large amount of muscle progenitor cell proliferation occurs; and the adult 24-month-old stage, during which myofibers are well established (Hocquette, 2010; Yue et al., 2017). Considering the differential expression of RNAs from these two stages, these RNAs might be associated with myogenesis, particularly lncRNAs and miRNAs that are known to have more developmental stage-specific expression patterns than protein-coding genes (Guttman and Rinn, 2012). According to the study, a series of exquisitely regulated and orchestrated changes happened at multiple levels including mRNA transcription and translation, protein stability, and degradation from the embryonic to the adult stage (Bismuth and Relaix, 2010). CeRNA, a well-known regulatory mechanism sets up an extensive regulatory network among the transcriptome, in which lncRNAs have been proposed to sponge miRNAs and thereby regulate other transcripts containing MREs (Denzler et al., 2016). In spite of their low abundance and/or nuclear localization, thousands of lncRNAs possess developmental stage-specific expression patterns and localizations (Mercer and Mattick, 2013; Koufariotis et al., 2015). Based on this reasoning, a global triple network was visualized in our study to predict potential lncRNA-mediated networks in myogenesis (Shannon et al., 2003).

In this study, natural ceRNA crosstalk interactions, with prominent expression shifts under the two stages, were screened bioinformatically and topological properties of the network verified more accepted ceRNA activity of lncRNAs (Gursoy et al., 2008). These observations suggested that the constructed ceRNA network could serve as a powerful prediction tool to explore the lncRNAs’ ceRNA function and role in bovine skeletal muscle development. Accumulated research showed that lncRNA ceRNAs have been implicated in myogenesis: lincMD1 controls muscle differentiation in human and mouse myoblasts directly by competing for miR-133 and miR-135, regulating the expression of MAML1 and MEF2C mRNAs, respectively (Cesana et al., 2011); acting as a molecular sponge, H19 lncRNA, which is highly expressed in the developing embryo and adult muscle, was found to bind to let-7 and inhibits its myogenesis function (Kallen et al., 2013); Zhu et al. (2017) reported that as a ceRNA for microRNA-125b, lnc-mg facilitates myogenesis with *IGF2*; while current reports showed that lnc133b/miR-133b/*IGF1R* axis is a potential pathway for promoting satellite cell proliferation and repressing their differentiation through the ceRNA mechanism (Jin et al., 2017); and Linc-smad7 was reported to promote myoblast differentiation and muscle regeneration via sponging miR-125b (Song et al., 2018). These findings indicate that individual lncRNAs may be potent natural miRNA sponges in certain settings, and lncRNA-associated ceRNA crosstalk will likely shift under different specific conditions.

To further research, we used an efficient way to infer the potential function of lncRNAs by studying miRNAs and/or mRNAs annotated functions (Wang et al., 2016; Zhou et al., 2016). In such a scenario, the functional enrichment analysis of 69 co-expression mRNAs in the network were performed including GO and KEGG pathway analysis. It was found that significant GO categories and pathways of DEMs were mainly enriched in myogenesis, suggesting the possible roles of these lncRNAs in bovine skeletal muscle development. Intriguingly, associated genes in myogenesis-related GO terms are highly consistent with those in a Wnt signaling pathway, which plays an essential role during embryonic muscle development and in the maintenance of adult skeletal muscle homeostasis (von Maltzahn et al., 2012). For instance, *APC* dampens canonical Wnt signaling to regulate mouse muscle stem cell proliferation and quiescence (Parisi et al., 2015); *CTNNB1*, the coding gene of β-catenin which is a key component of the canonical Wnt signaling pathway, was reported to alter the expression of some myogenic markers and downregulates myogenesis by regulating the expression of PPARG (Gustafsson et al., 2002; Okamura et al., 2009); as a Wnt antagonist, SFRP2 is upregulated during injury regeneration and exerts a differentiation inhibitory action on fibroblasts (Zhao and Hoffman, 2004; Descamps et al., 2008). However, previous *in vitro* studies have revealed that miR-199a-5p was affects myoblast differentiation by targeting several myogenic cell proliferation and differentiation regulatory factors of the Wnt signaling pathway, such as FZD4, JAG1, and WNT2 (Alexander et al., 2013). Functional analysis has suggested the possible correlation between the lncRNA-associated ceRNA crosstalk and the effects of Wnt on skeletal muscles.

The limitations of the network should be acknowledged. Early screening of differential expressed mRNAs was so strict that many myogenesis-related mRNAs were missed. Thus, Gene Set Enrichment Analysis was carried out to identify the associated gene effect from the embryonic to the adult stage, and the results determined a significant correlation between the adult high expression and the myogenesis-related gene set. Interestingly, our network also showed the mRNA-upregulated expression. Previous experiments suggested that *SMAD4*, whose protein levels increased dramatically in myoblasts of aged mice, is directly down-regulated by miR-431 and miR-26a to promote muscle differentiation and regeneration (Dey et al., 2012; Lee et al., 2015); *SRF* expression is up-regulated after birth and subsequently stabilizes in adulthood, and *SRF* inhibits muscle cell proliferation and differentiation *in vitro* and *in vivo* (Wang et al., 2002; Li et al., 2005). Accordingly, we infer that some mRNAs upregulated at the adult stage may exert a differentiation inhibitory action on fibroblasts, which are regulated by signaling pathways such as the Wnt signaling (Descamps et al., 2008).

To our knowledge, this is the first study that constructs a lncRNA-associated ceRNA network in Chinese Qinchuan bovine skeletal muscle development. Many miRNA-target gene pairs (miR-335-*RB1*, miR-122-*SRF*, miR-377-*SOD1*) were literately evaluated, and the validation of differentially expressed RNAs by qPCR further increases the credibility of our network (Scarola et al., 2010; Wang W. et al., 2015; Zeng et al., 2015). However, it has been suggested that, the ceRNA effectiveness was influenced by various factors including miRNA- and ceRNA- abundance, RBPs (RNA binding proteins) and subcellular localization, as well as the number and binding affinities of the MREs (Mukherji et al., 2011; Wee et al., 2012; Liu and Miao, 2016), so further experimental studies should be conducted to uncover the lncRNAs ceRNA function in bovine skeletal muscle development as a next step.

## Materials and Methods

### Ethics Statement

The animal care and experiments were conducted according to the Administration of Affairs Concerning Experimental Animals (Ministry of Science and Technology, 2004) China and approved by the Institutional Animal Care and Use Committee (College of Animal Science and Technology, Northwest A&F University, China). All samples (heart, liver, spleen, lung, kidney, and longissimus muscle) from Qinchuan cattle at the embryonic stage (90 days) and the adult stage (24-month-old) were collected at Shannxi Kingbull Animal Husbandry Co., Ltd. (Baoji, China). Adult animals were humanely killed where necessary, to ameliorate suffering and were not fed the night before they were slaughtered.

### High-Throughput Sequencing Data

The sequence-based data were obtained from our high-throughput sequencing results, including lncRNA (Supplementary Table S5), miRNA (Supplementary Table S6), and mRNA (Supplementary Table S7) libraries from Chinese Qinchuan bovine longissimus muscles in two developmental stages (embryonic 90-day and adult 24-month-old). In the process, six RNA samples, that passed the quality control, were pooled to obtain an “averaged” transcriptome at each developmental stage (mRNAs were not “averaged”), and the databases were based on the platform of Solexa technology (Beijing Genomics Institute, China) and Illumina HiSeq 2500 Technology (LC Sciences, Houston, TX, United States).

### Data Processing

The DEMis, DELs, and DEMs between two developmental stages were selected according to the | log_2_FC|≥ 4(| log_2_FC|≥ 2 for DEMs) and *P*-values < 0.001, which were used to control the number of differentially expressed RNAs within a manageable but useful range, not too many or too few. Subsequently, the DEMis were handled using the LncACTdb (LncRNA-associated Competing Triplets DataBase) to provide competition based lncRNA–miRNA–mRNA interactions, from which we obtained the DEMis-DEMs pairs (Wang P. et al., 2015). Then the DEMis-DELs pairs were predicted using the RNAhybrid program based on the MFE (minimum free energy) of miRNA–lncRNA duplexes (Rehmsmeier et al., 2004). Data for these interactions were downloaded from miRBase (Kozomara and Griffiths-Jones, 2014) and NONCODE (Zhao et al., 2016). In order to acquire high-quality target lncRNAs, DELs that had perfect nucleotide pairing between the 2nd and 8th positions of the 5’end of DEMis sequences were selected. Finally, the DEMis-DEMs pairs were merged with the DEMis-DELs pairs to obtain the co-expression RNAs which were used to construct and visualize the network.

### Construction and Analysis of the lncRNA–miRNA–mRNA Network

The lncRNA–miRNA–mRNA network was constructed and visualized using Cytoscape software based on the ceRNA theory (Shannon et al., 2003). Here, nodes and edges were used to represent large biological data in an intuitive way, in which each node represents a biological molecule, the edges stand for the interactions between nodes, and the node degrees indicating the number of edges linked to a given node were calculated to exploit the hub nodes that possess essential biological functions (Kohl et al., 2011). To explore the structure and feature of the lncRNA–miRNA–mRNA competing triplets, a network analysis was performed using a Cytoscape plug-in called “Network Analyzer” (Assenov et al., 2008). Topological parameters like DC, BC, and CC, which are standard measures of centrality in a network, were assessed here (The DC is defined as the number of links incident upon a node, the BC for each node is calculated as the number of these shortest paths that pass through the node, and the CC of a node is the total of the length of the shortest paths between the node and all other nodes in the network).

### Functional Enrichment Analysis

In order to assess functional enrichment in the co-expression competing triplet, Cytoscape plug-in ClueGO was used to constitute the interaction network based on the GO and KEGG database (Mlecnik et al., 2017). In this section, functional annotation of the 69 co-expression mRNAs were performed^1^ to infer the function of each co-expression lncRNA, and the statistical test used for the enrichment was based on the two-sided hypergeometric test with a Bonferroni step-down correction and kappa score of 0.4.

### GSEA

Gene Set Enrichment Analysis was performed based on our mRNA-seq data using the GSEA software version 3.0^2^. The mRNA-seq data were pre-ranked to form a gene-list according to their expression levels between two developmental stages. Following this, certain gene sets from MSigDB (H and C3 MIR gene set) were mapped to the pre-ranked gene-list to calculate the ES (enrichment score), in which 1000 permutations were used to calculate significance, and a certain gene set with FDR < 0.01 was considered significant.

### Total RNA Extraction and Quantitative Real-Time PCR Analysis of RNAs

Using Trizol reagent (TakaRa; Japan), total RNA was extracted from Chinese Qinchuan bovine tissues. A total of 1000 ng total RNA was reverse transcribed into cDNA with the PrimeScript RT Kit (Takara), and miRNA-specific stem-loop primers (Supplementary Table S8) were used for reverse-transcribed cDNA of miRNAs. For RNA quantification, all measurements were replicated three times in a Bio-Rad master cycler using SYBR Green II Master Mix Reagent Kit (Takara) following the manufacturer’s instructions. qPCR primers are shown in Supplementary Table S8, and U6 (for miRNA) and GAPDH were used as the internal control for normalization of the data. The expression levels of lncRNAs, miRNAs and mRNAs were calculated using the 2^−ΔΔCt^ method.

## Data Availability

The datasets generated for this study can be found in NCBI, SRR1818309, SRR1818415, and SRR1818416.

## Author Contributions

HC conceived the study idea and designed the work. BY, HL, MeL, JW, and MiL analyzed and interpreted the data. BY wrote the manuscript. BH provided partial samples. CL revised the manuscript critically. All authors read and approved the final manuscript.

## Conflict of Interest Statement

The authors declare that the research was conducted in the absence of any commercial or financial relationships that could be construed as a potential conflict of interest.

## Footnotes

1 http://www.cytoscape.org/

2 http://www.broadinstitute.org/gsea
